# Network-Based Prediction and Analysis of HIV Dependency Factors

**DOI:** 10.1371/journal.pcbi.1002164

**Published:** 2011-09-22

**Authors:** T. M. Murali, Matthew D. Dyer, David Badger, Brett M. Tyler, Michael G. Katze

**Affiliations:** 1 Department of Computer Science, Virginia Polytechnic Institute and State University, Blacksburg, Virginia, United States of America; 2 Applied Biosystems, Foster City, California, United States of America; 3 Virginia Bioinformatics Institute, Virginia Polytechnic Institute and State University, Blacksburg, Virginia, United States of America; 4 Department of Microbiology, University of Washington, Seattle, Washington, United States of America; Utrecht University, Netherlands

## Abstract

HIV Dependency Factors (HDFs) are a class of human proteins that are essential for HIV replication, but are not lethal to the host cell when silenced. Three previous genome-wide RNAi experiments identified HDF sets with little overlap. We combine data from these three studies with a human protein interaction network to predict new HDFs, using an intuitive algorithm called SinkSource and four other algorithms published in the literature. Our algorithm achieves high precision and recall upon cross validation, as do the other methods. A number of HDFs that we predict are known to interact with HIV proteins. They belong to multiple protein complexes and biological processes that are known to be manipulated by HIV. We also demonstrate that many predicted HDF genes show significantly different programs of expression in early response to SIV infection in two non-human primate species that differ in AIDS progression. Our results suggest that many HDFs are yet to be discovered and that they have potential value as prognostic markers to determine pathological outcome and the likelihood of AIDS development. More generally, if multiple genome-wide gene-level studies have been performed at independent labs to study the same biological system or phenomenon, our methodology is applicable to interpret these studies simultaneously in the context of molecular interaction networks and to ask if they reinforce or contradict each other.

## Author Summary

Medicines to cure infectious diseases usually target proteins in the pathogens. Since pathogens have short life cycles, the targeted proteins can rapidly evolve and make the medicines ineffective, especially in viruses such as HIV. However, since viruses have very small genomes, they must exploit the cellular machinery of the host to propagate. Therefore, disrupting the activity of selected host proteins may impede viruses. Three recent experiments have discovered hundreds of such proteins in human cells that HIV depends upon. Surprisingly, these three sets have very little overlap. In this work, we demonstrate that this discrepancy can be explained by considering physical interactions between the human proteins in these studies. Moreover, we exploit these interactions to predict new dependency factors for HIV. Our predictions show very significant overlaps with human proteins that are known to interact with HIV proteins and with human cellular processes that are known to be subverted by the virus. Most importantly, we show that proteins predicted by us may play a prominent role in affecting HIV-related disease progression in lymph nodes. Therefore, our predictions constitute a powerful resource for experimentalists who desire to discover new human proteins that can control the spread of HIV.

## Introduction

Conventional high-throughput antiviral discovery often targets the activities of specific viral enzymes. These approaches have been ineffective in stemming the emergence of drug-resistant variants, especially in the face of rapidly-mutating RNA viruses. One powerful yet under-explored avenue is the evolutionarily resilient nature of host proteins. Viral pathogens are parasitic in nature owing to their limited genomes. In principle, disruptions to host-pathogen interactions would impede the propagation of pathogens. The recent identification of HIV dependency factors (HDFs) or “host cellular factors” highlights this point [1], [2], [3]. HDFs represent a class of host proteins that are essential for HIV replication, but are not lethal to the host cell when silenced. By measuring levels of viral protein expression or production of infectious viral particles in human cells after knocking down individual genes using RNA interference (RNAi), these studies search for human genes that are required by HIV. Such studies have also been performed for other viruses and bacteria pathogenic to humans [4], [5], [6], [7], [8]. HDFs not only provide critical insights into HIV pathogenesis by helping to identify potential mechanisms for manipulation of host pathways, but may also have the potential to serve as therapeutic targets.

The studies conducted by Brass *et al.* [1], Konig *et al.* [2], and Zhou *et al.* [3] identified 275, 296, and 375 HDFs, respectively. The Brass and Konig sets had an overlap of 13 proteins, the Konig and Zhou sets had an overlap of 10 proteins, while the Brass and Zhou sets had 17 common proteins. One potential reason for the small overlap is that the experiments were performed in different cell lines; the Brass and Zhou studies used HeLa cells while the Konig study used HEK293T cells. The small overlaps could also arise from differences in the HIV strains used, the assay time post-infection, the procedures used to measure infection, and other approaches used to analyze experimental data [9], [10]. Although the three siRNA screens showed little overlap at the level of individual genes, Bushman *et al.* [10] found that similar Gene Ontology (GO) terms were enriched in the three gene sets. Interestingly, Konig *et al.* noted that 64 HDFs reported by Brass *et al.* directly interacted (via a physical interaction between proteins) with a confirmed HDF in their study. In support of this observation, Bushman *et al.* constructed a network of protein-protein interactions among HIV proteins and 2,410 host cell genes identified in the three siRNA screens and six other HIV-related studies. Dense clusters within this network contained multiple proteins identified in two or more siRNA screens and were enriched in processes and complexes such as the proteasome and the mediator complex, which are known to be associated with HIV replication. In a related study, Wuchty *et al.* [11] found that HDFs and human proteins that interact with HIV also appeared in dense clusters. The proposed that such protein groups may serve as “infection gateways” that enable the virus to control specific human cellular processes. They also noted that transcription factors and protein kinases mediated indirect interactions between HDFs and viral proteins. Macpherson *et al.* [12] performed a complementary analysis. Starting from known human-HIV protein-protein interactions (PPIs), they used biclustering to identify sets of human proteins that participated in the same types of interactions with HIV proteins. They evaluated the functional information in each bicluster and further grouped the human proteins in biclusters into higher-level subsystems. By overlapping these subsystems with HDFs, they characterized host systems that were perturbed by HIV-1 infection and identified patterns of human-HIV PPIs that correlated to these perturbations.

We took these analyses as our starting point, since they suggested that the three siRNA genomic screens may be incomplete and that there are potentially many HDFs yet to be discovered. In particular, we hypothesized that the proximity of experimentally-detected HDFs within the human protein-protein interaction (PPI) network can be fruitfully exploited by machine-learning algorithms to predict novel HDFs. We treated the computational problem of predicting HDFs as an instance of semi-supervised learning: we combined HDFs identified by Brass *et al.*, Konig *et al.*, or Zhou *et al.* (positive examples formed by the union of these three sets) with non-HDFs (negative examples, see “Data and Algorithms” for details) in the context of a human PPI network. The other proteins in this network constituted the unknown examples. We used an intuitive graph-theoretic approach that we call SinkSource and other algorithms published in the literature [13], [14], [15] to predict undiscovered HDFs. Our results, along with those of other studies [10], [11], [12], suggest that many HDFs are yet to be discovered and that they have potential value as prognostic markers to determine pathological outcome and the likelihood of AIDS development.

## Results/Discussion

The SinkSource algorithm can be understood via the following physical analogy. We consider the PPI network to be a flow network. Here, each edge is a pipe and its weight denotes the amount of fluid that can flow through the pipe per unit time. Each node has a reservoir of fluid. We maintain the level of the reservoir at each HDF at 1 unit and at each non-HDF at 0 units. We let fluid flow through this network. At equilibrium (when the amount of fluid flowing into each node is equal to the amount flowing out), the reservoir height at each node denotes our confidence that the node is an HDF. Our approach is reminiscent of the FunctionalFlow algorithm [14] developed for predicting gene functions, with one crucial difference. The FunctionalFlow algorithm does not use negative examples, permitting the reservoir level at a node to increase without bound. Hence, the algorithm stops after a user-specified number of phases. In contrast, our algorithm will converge to a unique solution.

We applied seven prediction algorithms to the HDF data in the context of a human PPI network integrated from seven public databases [16], (see “Data and Algorithms”). The algorithms were the SinkSource algorithm; a variant called SinkSource+ that does not need negative examples; the commonly-used guilt-by-association approach, both with and without negative examples (called Local and Local+ in this work); a method based on Hopfield networks [13]; the FunctionalFlow algorithm [14]; and another flow-based approach called PRINCE [15]. Guilt-by-association, Hopfield networks, and FunctionalFlow have been proposed to address the problem of gene function prediction. PRINCE is an approach to prioritize disease-related genes; we selected PRINCE since it outperformed many other methods for predicting disease related genes, including cluster and neighborhood based algorithms. We applied the algorithms to four sets of positive examples: the HDFs in the Brass *et al.* study (B), the HDFs in the Konig *et al.* study (K), the HDFs in the Zhou *et al.* study (Z), and the union of these three sets (BKZ). We restricted these sets to those proteins that participated in at least one interaction in the human PPI network. We used an unweighted version of the network for all results below.

### Combining the Brass, Konig, and Zhou datasets improves cross-validation results

**Figure 1:**
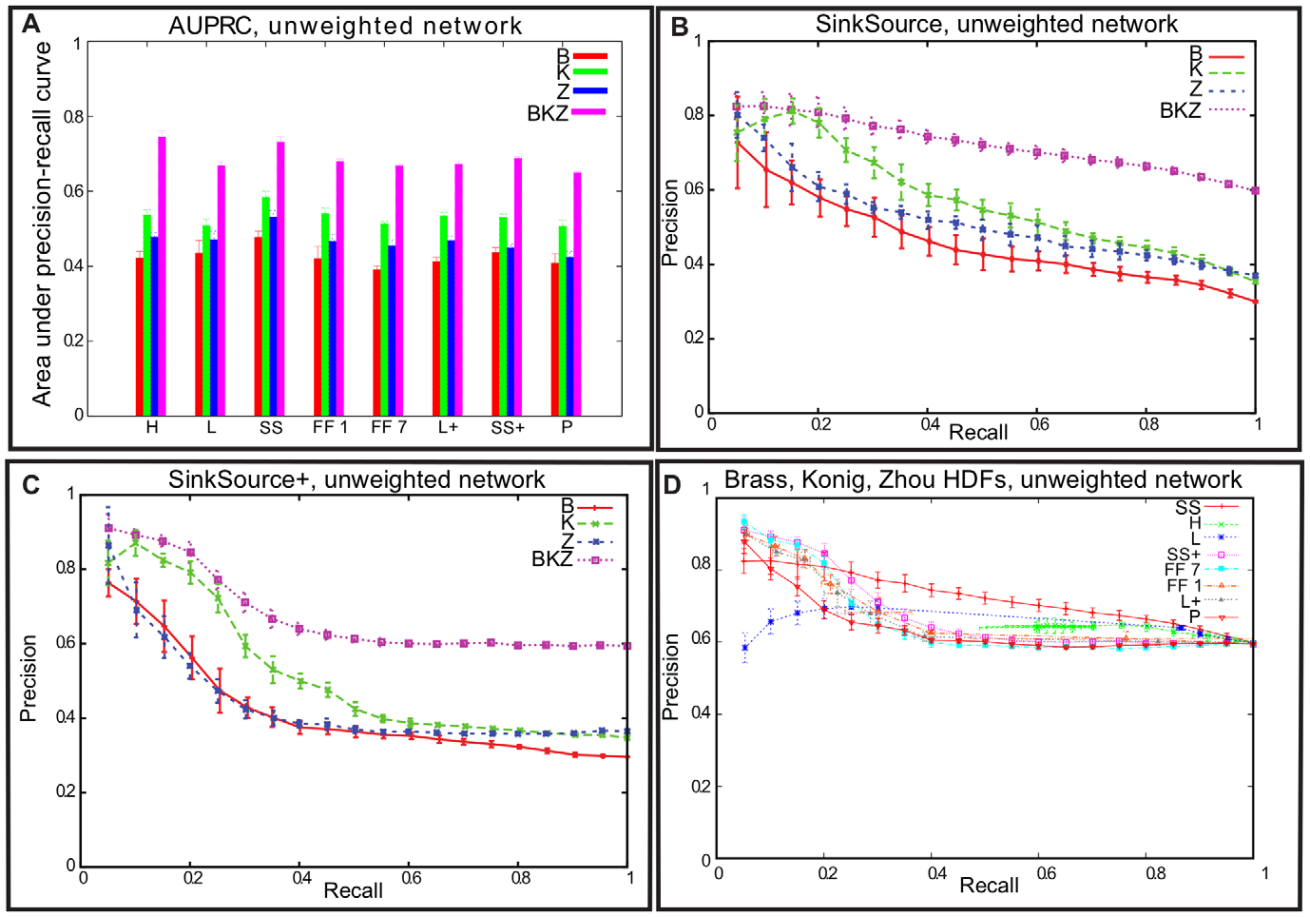
Cross validation results on the unweighted human PPI network. (a) Histograms of area under precision-recall curve for all algorithm-dataset combinations. Each group of vertical bars corresponds to one algorithm. Error bars indicate one standard deviation from the mean, computed over 10 independent runs of 2-fold cross validation. Algorithm abbreviations: Hopfield (H), Local (L), SinkSource (SS), FunctionalFlow with 1 phase (FF 1), FunctionalFlow with 7 phases (FF 7), Local without negative examples (L+), SinkSource without negative examples (SS+), and PRINCE (P). Dataset abbreviations: Brass (B), Konig (K), Zhou (Z), Brass or Konig or Zhou (BKZ). (b) Precision-recall curves for the SinkSource algorithm on the four datasets. At each value of recall, error bars indicate one standard deviation in the value of precision. (c) Precision-recall curves for the SinkSource+ algorithm on the four datasets. (d) Precision-recall curves for all algorithms on the BKZ dataset.

Figure 1 displays the results of two-fold cross validation for the six algorithms tested on four datasets. Two-fold cross validation involves splitting the positive and negative examples into two halves, and using each half to make predictions for the genes in the other half. We used two-fold cross validation since we felt it better mimics our state of knowledge of HDFs than the more commonly used five-fold or 10-fold cross validations. We averaged the results over 10 independent runs for each algorithm-dataset combination. For each algorithm, it is evident from Figure 1(a) that the area under the precision-recall curve (AUPRC) value for the BKZ dataset is larger than the values for the B, K, or Z datasets. It is also clear that these results are robust to the randomization inherent in cross validation: the largest standard deviation in the AUPRC values is 0.033 (as indicated by the error bars in Figure 1(a) and data in Table S1). Figure 1(b) displays the precision-recall curve for SinkSource on the four datasets and Figure 1(c) shows the results for SinkSource+. The results for SinkSource+ were obtained with an internal parameter λ set to a value of 1 (see “Other Algorithms” for the role played by this parameter in the SinkSource+ algorithm). In each figure, we observed that the curve for the BKZ dataset dominated the other three curves at most values of recall. This result is consistent with the expectation that the Brass, Konig, and Zhou studies did not discover all true HDFs, and that combining the three sets provides a better coverage of the true HDF universe. We also noted that the variation in precision (indicated by the error bars in Figure 1(b) and Figure 1(c)) decreases with increasing recall, suggesting that high confidence predictions are more subject to variation than low confidence predictions. Finally, Figure 1(d) compares the performance of all seven algorithms on the BKZ dataset. Three of the algorithms that do not use negative examples (Local+, SinkSource+, and Functional Flow with 1 and with 7 phases) achieved higher precision values than the other algorithms for values of recall less than 20%. However, SinkSource has the best performance for values of recall greater than 20%. PRINCE, the fourth algorithm that did not use negative examples, had uniformly lower precision than SinkSource+. Its precision was superior to that of SinkSource for values of recall less than 10%. To obtain the results for PRINCE, we used 0.8 for the value of an internal parameter α, since PRINCE achieved the highest precision values for this setting of α (see “Other Algorithms” for the role played by this parameter in the SinkSource+ algorithm). Furthermore, the precisions of the algorithms that do not use negative examples dropped considerably beyond a recall of 20% (beyond 10% in the case of PRINCE). We believe that this performance drop is caused by an undue influence of positive examples, resulting in many false positives. The performance of FunctionalFlow did not vary much with an increase in the number of phases (see Figure S1). The performance of SinkSource+ was independent of the parameter λ (see Figure S2), as was the performance of PRINCE with respect to the parameter α (see Figure S3). We also noted that the AUPRC values for the BKZ dataset were 0.67 for Local, Local+, and for FunctionalFlow with 7 phases, 0.65 for PRINCE, 0.69 for SinkSource+, 0.73 for SinkSource, and 0.74 for Hopfield. There is a difference of 11% between the AUPRCs of the worst performing algorithms (0.67) and the best performing algorithm (0.74). The results for weighted versions of the network did not substantially differ from those for the unweighted network (see Figure S4 and Table S2).

The SinkSource algorithm achieved a precision of 81% at 20% recall. The precision dropped only to 70% at a recall of 60%. The corresponding precisions for SinkSource+ were 85% and 60%. Although the Hopfield network algorithm achieved an AUPRC of 0.74, we observed that the smallest recall value attained by the algorithm was 60%, since the algorithm assigned a confidence of either 1 or −1 to a large number of predictions. We concluded that the Hopfield network algorithm was not a good choice for prioritizing predictions for further experimental analysis.

It is surprising that the very simple guilt-by-association algorithms (Local+ and FunctionalFlow with one phase) perform nearly as well as more sophisticated methods (FunctionalFlow with 7 phases, Hopfield, PRINCE, and SinkSource) that attempt to optimize predictions by taking into account constraints imposed by the entire protein interaction network. However, across 10 runs of cross validation, both Local+ and FunctionalFlow with one phase showed higher variation in precision and recall than the other algorithms (see Figure S5). Therefore, these two algorithms are likely to be more susceptible to missing or erroneous information.

Based on these results, we concluded that SinkSource+ and SinkSource were the two best algorithms for predicting HDFs. When high precision is required, SinkSource+ is superior to SinkSource. Thus, the predictions made by SinkSource+ might be the most suitable as the basis for detailed experimental studies of candidate HDFs. In the rest of the paper, we focus on the results obtained by the SinkSource+ and SinkSource algorithms.

### SinkSource+ and SinkSource make overlapping predictions

We compared how many predictions SinkSource+ and SinkSource made at confidence values that correspond to approximately 80% precision after cross validation. SinkSource+ achieved a precision of 85% (and a recall of 20%) at a confidence of 0.5. The corresponding numbers for SinkSource were a confidence of 0.71 at a precision of 81% (and a recall of 20%). To further compare the two algorithms, we computed the overlaps in their predictions for different cutoffs on the confidence values. Specifically, we computed the *k* highest confidence genes predicted by SinkSource+ and the *k* highest-confidence genes predicted by SinkSource, and measured the Jaccard coefficient of the pair of gene sets, for different values of *k* in increments of 100. Figure S6 demonstrates that the overlap between the predictions of the two algorithms is at least 0.34 up to the first 2000 predictions, with peaks at around 300 and 1000 predictions. These results are consistent with the relatively low recall (20–40%) predicted for the two algorithms at this level of precision. The data suggest that approximately half of the predictions may be ranked differently by the two algorithms. Predictions made by SinkSource+ for different values of the parameter λ did not vary much in their ranking (see Figures S7 and S8).

On the basis of these comparisons, we identified a set of high confidence predictions composed of the 1000 top-ranked predictions from SinkSource+ and from SinkSource respectively. These two sets contained 606 predictions in common and comprised a total of 1394 proteins in addition to the 908 BKZ HDFs. At the confidence levels of the 1000 SinkSource and SinkSource+ predictions, the precisions with two-fold cross validation are 88% and 81% respectively, suggesting that these predictions are relatively reliable. The corresponding recalls with two-fold validation are roughly 17% and 15% respectively, suggesting that these predictions are quite conservative.

In the rest of the paper, we use the phrases “BKZ HDFs”, “SS+ predicted HDFs”, and “SS predicted HDFs” to distinguish between the HDFs identified by one or more of the three siRNA screens [1], [2], [3], the HDFs predicted by SinkSource+, and the HDFs predicted by SinkSource, respectively. We extensively evaluated the predicted HDFs by comparing them to each other and to BKZ HDFs in terms of their functional annotations, interactions with HIV proteins, clustering with the PPI network, and role in disease pathogenesis. We based these evaluations on additional datasets that we did not use for predicting HDFs. Specifically, the new datasets we used were (i) Gene Ontology (GO) annotations for human proteins, (ii) interactions between HIV and human proteins, and (iii) gene expression data from two non-human primate species following infection with SIV. Hence, the analyses described below constitute independent evaluation of the relevance of our predictions to HIV infection and disease progression.

### Predicted HDFs are enriched in HIV-related GO terms

We summarized the functional roles of predicted HDFs by asking which GO terms were enriched in the HDFs, and whether any terms were considerably enriched in predicted HDFs but not in BKZ HDFs. We used the FuncAssociate software [23] for this purpose, since it can take ordered lists of genes as input, in which case it finds and utilizes the set of top-ranked genes displaying the greatest enrichment. FuncAssociate adjusts for multiple hypotheses testing by computing an experiment-wise *p*-value. Note that FuncAssociate operates solely on the ranked list of genes and the GO annotations. It does not utilize a network. (See “Methods” for details.) We invoked FuncAssociate with three inputs: (a) the unordered set of BKZ HDFs, (b) the SS+ predicted HDFs, ordered by confidence, and (c) the SS predicted HDFs, also ordered by confidence. We used default values of all other parameters used by FuncAssociate. FuncAssociate reported 52 GO terms as being enriched in BKZ HDFs with an adjusted *p*-value of 0.05 or less and 199 GO terms as enriched in SS+ predicted HDFs. We identified three classes of terms (see Table S3). We note that FuncAssociate may report many related terms as enriched, due to the hierarchical nature of GO. Therefore, we also manually inspected the directed acyclic graph connecting the enriched terms in order to make the observations below.

- *49 GO terms enriched in both BKZ HDFs and SS+ predicted HDFs*: For the most part, these terms corresponded to the biological processes or complexes that were also identified by Bushman *et al.* [10]. These terms included the proteasome, transcription/RNA polymerase, the mediator complex, transcriptional elongation, and RNA binding and splicing. This recapitulation is not surprising since Bushman *et al.* identified these GO terms by searching for dense PPI subnetworks connecting BKZ HDFs and other HIV-related proteins. Proteins in such dense subgraphs are likely to be adjacent in the PPI network to proteins that are predicted to be HDFs with high confidence by our algorithms.
- *3 GO terms enriched in BKZ HDFs but not in SS+ predicted HDFs*: Three terms enriched only in BKZ HDFs were nucleocytoplasmic transporter activity, proteasome core complex, alpha-subunit complex, and Golgi apparatus. Except for Golgi apparatus, closely related terms were enriched in predicted HDFs.
- *413 GO terms enriched only in SS+ predicted HDFs*: Many GO terms were enriched only in SS+ predicted HDFs. Examples are GO terms corresponding to two protein complexes, the Ndc80 complex (GO:0031262) and MIS12/MIND type complex (GO:0000444). Both terms were enriched only in predicted HDFs with a *p*-value of 0.002. All four components of the Ndc80 complex (NDC80, NUF2, SPC24, and SPC25) and all four components of MIS12/MIND type complex (DSN1, MIS12, NSL1, and PMF1) occurred within the top 275 predictions made by SinkSource+. Both complexes are part of the kinetochore and play important roles in forming stable kinetochore-microtubule attachments. Retroviruses such as HIV hijack microtubules in order to cross the cytoplasm into the nucleus and to allow HIV gene products to return to the cell surface [24]. Although the Ndc80 and MIS12/MIND type complexes have not been directly implicated in the HIV life cycle, they represent new candidates for involvement in HIV movement through the host cell cytoplasm.

The trends were similar for the HDFs predicted by SinkSource (data not shown). Therefore, we compared the FuncAssociate results for SS+ predicted HDFs and for SS predicted HDFs in a similar manner. We only considered GO terms enriched with an adjusted *p*-value of 0.05 or less. As shown in Table S4, 280 GO terms were enriched in both sets of predictions, 182 GO terms were enriched only in SinkSource+ predictions, and 25 GO terms were enriched only in SinkSource predictions. The 280 common terms were related to processes such as RNA splicing (GO:0008380), translation initiation (GO:0003743), and oxidative phosphorylation (GO:0003743) and complexes such as the proteasome (GO:0003743), the kinetochore (GO:0000776), and the nuclear pore (GO:0005643); we discuss their relevance to HIV when we discuss clusters in the PPI network below (See “PPI Clusters Spanned by BKZ HDFs and Predicted HDFs Are Exploited by *HIV*”). The 182 GO terms enriched only in SinkSource+ predictions included the Ndc80 complex and MIS12/MIND type complex (mentioned above), apoptosis (including its induction and regulation) (GO:0006915, GO:0006917, and GO:0042981), and specializations of terms enriched in both sets of predictions. Among the 25 GO terms enriched only in SinkSource predictions, there were 12 GO terms whose specializations or near neighbors (in the GO directed acyclic graph) were enriched in SinkSource+ predictions. Each of the remaining 13 GO terms enriched only in SinkSource predictions were closely related to the assembly of glycosylphosphatidylinositol (GPI) anchors (GO:0006506). Based on these results, we concluded that, for the most part, similar functions were enriched in HDFs predicted by SinkSource+ and by SinkSource.

### SS+ predicted HDFs interact with HIV proteins to a statistically-significant extent

Bushman *et al.* observed that each of the Brass, Konig, and Zhou HDF sets were statistically significantly enriched with human proteins that interact with HIV proteins (as reported in the NCBI HIV interaction database [25]). We hypothesized that predicted HDFs might be significantly enriched with HIV interactors. Accordingly, for each algorithm, we selected the *k* top ranking predictions made by that algorithm, for different values of *k* starting at 100 and in increments of 100, computed the overlap of each set of predictions with the human proteins that interact with HIV, estimated the statistical significance of the overlap using the one-sided version of Fisher's exact test, and adjusted the *p*-values to account for testing multiple hypotheses [26]. The overlap fraction for SS+ predicted HDFs peaked at 26% (79 of the top 300 predicted HDFs interact with HIV proteins, *p*-value 2.1×10^−7^), better than the BKZ HDFs of which 20% (109 proteins, *p*-value 9.11×10^−6^) interacted with HIV proteins. The trend for SS predicted HDFs was mixed: the overlap ratio was as high as 17.5% (70 of the top 400 predictions interact with HIV proteins), slightly less than the BKZ HDFs, but in no case was the enrichment statistically significant. These results suggest that SinkSource+ HDF predictions are dominated by proteins that lie close to BKZ and HIV proteins in the joint HIV-human PPI network, whereas the SinkSource predictions are dispersed further away. We discuss specific SS+ predicted HDFs that interact with HIV in the context of MCODE clusters below.

### PPI clusters spanned by BKZ HDFs and SS+ predicted HDFs are exploited by HIV

The cross validation analysis suggested that HDFs are not randomly located in the human PPI network. Rather, HDFs are closer to each other within the PPI network than to the negative examples. Therefore, in order to better understand how BKZ HDFs and SS+ predicted HDFs are related to each other, we computed the subnetwork of PPIs spanned by these two sets of genes. We applied a modified version of the well-known MCODE [27] graph clustering algorithm to this sub-network (see “Modifying MCODE to Compute PPI Clusters”). The network contained 1,562 proteins and 30,855 PPIs. MCODE identified 41 clusters of varying sizes containing a total of 829 proteins and 16,721 PPIs. Table 1 contains statistics on the 10 clusters with the largest number of PPIs computed by MCODE. Using the one-sided version of Fisher's exact test, we checked the overlap of each of the 42 clusters with BKZ HDFs. Only eight clusters had overlaps that were statistically significant, as shown in Table S5. Table S6 contains a list of BKZ HDFs and HDFs predicted by SinkSource+, annotated with MCODE cluster membership and information on interaction with HIV proteins. Table S7 lists the human PPIs in each MCODE cluster.

**Table 1:** Statistics on the 10 clusters with the largest number of PPIs reported by MCODE.

Ranking by #PPIs	#proteins	#PPIs	Density	Median rank	Minimum rank	Maximum rank	#HIV interactors	#BKZ HDFs
1	112	5684	0.91	44	1	210	34	33
2	108	4701	0.81	408	164	588	11	12
3	60	1770	1	222	152	419	5	0
4	57	1596	1	138	107	230	2	10
5	29	331	0.81	507	452	659	6	3
6	24	273	0.99	812	730	978	4	1
7	26	264	0.81	76	31	178	2	11
8	20	182	0.96	141	80	239	35	20
9	37	304	0.46	264	69	584	46	9
10	56	443	0.29	854	779	998	11	0

We computed GO terms enriched in all clusters. Table 2 contains statistics on highly enriched GO terms in the 10 most highly-connected clusters discovered by MCODE. Among the top 10 clusters, only clusters #1, #4, #7, #8, and #9 have statistically significant overlaps with BKZ HDFs (see Table S5). The fraction of BKZ HDFs is small in clusters #1, #4, and #9, so we reasoned that any functions enriched in these clusters would not be overly influenced by annotations of BKZ HDFs. In contrast, more than half the proteins in clusters #7 and #8 are BKZ HDFs; the functions enriched in these clusters are likely to annotate a number of BKZ HDFs. We now discuss the enriched functions in all clusters in Table 2. We focus our discussion on selected predicted HDFs contained within these clusters and present the support in the literature for the relevance of these HDFs to HIV pathogenesis.

**Table 2:** The ten clusters with the largest number of PPIs reported by MCODE and the functions that each is the most enriched in.

Ranking by #PPIs	#proteins	#HIV interactors	Highly enriched functions	*p*-value	#proteins with function	#BKZ HDFs	#BKZ HDFs with function
1	112	34	RNA metabolic process	1.4×10^−69^	107	33*	29
			Spliceosomal complex	2.7×10^−36^	52		14
2	108	11	Ribosome	7.1×10^−96^	75	12	0
			Translational elongation	9.5×10^−88^	75		0
3	60	5	Kinetochore	2.2×10^−42^	33	0	0
4	57	2	Respiratory chain	2.8×10^−80^	47	10*	9
			NADH dehydrogenase complex	2.9×10^−75^	34		6
5	24	4	small GTPase mediated signal transduction	1.6×10^−9^	21	3	1
6	29	6	DNA replication initiation	3.6×10^−14^	13	1	0
7	20	2	Transcription factor binding	3.4×10^−10^	13	11*	7
			Transcription initiation	5.3×10^−9^	12		6
8	60	35	Proteasome complex	6.8×10^−29^	18	20*	13
9	37	46	Proteasome complex	2.3×10^−33^	22	9*	0
10	39	11	MHC protein complex	9.2×10^−17^	10	0	0
			Cell cycle process	5.2×10^−7^	13		

Some columns are repeated from Table 1 for the sake of convenience.

*(in the column titled “#BKZ HDFS”) indicates that the overlap BKZ HDFs with clusters computed by MCODE is statistically significant at the 0.05 level.

#### Spliceosome

The most enriched function in cluster #1 is the biological process “RNA metabolic process” (*p*-value 1.4×10^−69^). As many as 52 proteins in this cluster are members of the spliceosome (*p*–value 2.7×10^−36^), which is a complex of specialized RNA and protein subunits that removes introns from a transcribed pre-mRNA segment. HIV interacts with several components of the spliceosome in order to stimulate transcription and viral production via the LTR [28], [29]. HIV has also been shown to inhibit the production of spliceosomal proteins as a mechanism to block downstream immune responses. 22 predicted HDFs and 14 BKZ HDFs in this cluster are known to interact with HIV. For example, the HIV VPR protein has been shown to hinder spliceosome assembly by interfering with the function of the SF3B2–SF3B4 host complex [30]; SinkSource+ predicts SF3B4 as an HDF with confidence 0.87 (rank 55). This disruption inhibits the correct splicing of several cellular pre-mRNAs, including β-globin and immunoglobulin M (IgM). IgM has an important role as both a regulator of the immune system and as an inhibitor of apoptosis. Blocking IgM production may allow the virus to inhibit an immune response and to activate cell death, phenomena that have been linked to the progression of HIV infection [31] High-ranking predicted HDFs with known HIV interactions that are members of the spliceosomal complex include the small nuclear ribonucleoproteins SNRPB, SNRPB2, SNRPD1, and SNRPD2. The HIV TAT protein interacts with SNRPD2 (predicted with a confidence of 0.87 and rank of 59 by SinkSource+) in order to stimulate transcription from the long terminal repeat (LTR) that acts as a switch to control the production of new viruses [28].

#### Translational elongation

Cluster #2 is enriched in the ribosome and in the biological process “translational elongation” with 75 of the 108 proteins in the cluster annotated with each of these terms (*p*-values 7.1×10^−96^ and 9.5×10^−88^, respectively). Bushman *et al.* [10] also identified a complex of 13 proteins involved in translation elongation. Our results substantially expand this complex. Among the proteins predicted by SinkSource+ that belong to this cluster, EIF2S1, EIF2S2, EIF2S3, EIF4E, EIF4G1, and EIF5B are known to interact with HIV molecules, supporting these predictions. TAR is a 5′-terminal hairpin in HIV-1 mRNA that binds viral Tat and several cellular proteins. Eukaryotic translation initiation factor 2 (EIF2) binds the TAR secondary structure in HIV-1 RNA [32], suggesting that TAR may be involved in the translation of viral mRNA. Another facet of HIV interaction with host translation elongation occurs in human CD4+ cells, where HIV-1 protease cleaves eukaryotic translation initiation factor EIF4G, thereby inhibiting host protein synthesis that is directed by capped mRNAs [33].

#### Kinetochore

Cluster #3 is highly enriched in the kinetochore (*p*-value 2.2×10^−42^). Other highly enriched GO terms include the MIS12/MIND type complex, the centromeric region of the chromosome, and the M phase of the mitotic cell cycle. The kinetochore is a multi-subunit protein complex that is located at the centromeric region of DNA. Microtubules connected to spindle poles attach themselves to the kinetochore. No BKZ HDFs are members of this cluster. However, five proteins in the cluster, KIF2C, BIRC5, PAFAH1B1, PPP1CC, and CDC20, are known to interact with HIV, supporting the validity of these HDF predictions. PAFAH1B1 (also known as LIS), a subunit of the platelet-activating factor acetylhydrolase, is a member of the kinetochore and the microtubule. The interaction of HIV-1 Tat protein with PAFAH1B1 may contribute to the effect of Tat on the distortion of microtubule formation [34], which in turn may induce apoptosis of T cells. In addition, this cluster may be related to HIV's utilization of the host cell cytoskeletal machinery to traffic from the cell membrane to the nucleus and vice-versa [24].

#### Mitochondrion

The most enriched GO term in cluster #4 is “respiratory chain” (*p*-value 2.8×10^−80^), with 47 of the 57 proteins in this cluster annotated with this term. Many of these genes are members of the NADH dehydrogenase complex (*p*-value 2.9×10^−75^), are involved in oxidative phosphorylation (*p*-value 2×10^−51^), and are localized to the mitochondrial membrane (*p*-value 1.8×10^−69^). Both the Brass and the Konig screens uncovered members of the NADH dehydrogenase complex, suggesting that HIV replication may involve the mitochondrial respiratory chain and the modulation of oxidative phosphorylation. The role played by host mitochondrial proteins in HIV-induced T-cell apoptosis has been extensively studied [35]. Recently, it has been shown that components of the mitochondrial oxidative phosphorylation system are differentially regulated in apoptotic T-cells that have been infected by HIV [36]. In eukaryotes, oxidative phosphorylation occurs in the electron transport chain in the mitochondrion. NADH dehydrogenase, a multi-subunit protein complex, is the first enzyme in this chain. The down-regulation of NDUFA6, a unit of the NADH dehydrogenase complex reported by both the Brass and Zhou screens, has been implicated in the induction of apoptosis in T cells by HIV [37]. SinkSource+ predicts NDUFS1, one of the units of this complex, as an HDF with confidence 0.82 (rank 185). Caspase cleavage of NDUFS1 has been shown to mediate disruption of mitochondrial function during apoptosis [38], suggesting that NDUFS1 may play a role in the induction of T cell apoptosis by HIV.

#### GTPase mediated signal transduction

Cluster #5 contains 24 proteins of which three are BKZ HDFs. 21 proteins in the cluster are involved in small GTPase mediated signal transduction, with a *p*-value of 1.6×10^−9^. Many proteins in the cluster belong to RAS family of proteins. Six proteins in the cluster, RHOB, RHOG, RAC2, RHOA, CDC42, and RAC1 are known to interact with HIV. Interactions of the small GTPases CDC42 and RAC1 with HIV protein Nef activates the p21-activated kinase 1 PAK1 [39], [40], a factor that is critical for efficient viral replication and pathogenesis.

#### DNA replication initiation

Of 29 proteins in cluster #6, 13 are annotated with the biological process “DNA replication initiation” (*p*-value 3.6×10^−14^). There are no BKZ HDFs in this cluster. However, four proteins in the cluster, CDC6, CDK2, PCNA, and RPA4 are known to interact with HIV proteins, suggesting the validity of these HDF predictions. Cyclin-dependent kinase 2 (CDK2) is a catalytic subunit of the cyclin-dependent protein kinase complex, whose activity is restricted to the G1-S phase, and which is essential for transition of the cell cycle from G1 to S phase. CDK2 phosphorylates HIV Tat protein, a step that is important for HIV-1 transcription [41], [42].

#### Mediator complex

Cluster #7 contains 20 proteins that are significantly annotated with the GO terms “Transcription factor binding” (3.4×10^−10^) and “Transcription initiation” (5.3×10^−9^). As many as 11 BKZ HDFs are members of this cluster. Almost all proteins in this cluster are subunits of the mediator complex. This complex enables transcription by connecting transcriptional activators to the RNA polymerase II transcriptional machinery [43], [44]. Bushman *et al.* [10] also identified this complex. They proposed that “changes in dosage in the mediator complex are not toxic to cells, but that Tat-activated transcription is extremely sensitive to mediator dosage.”

#### Proteasome

The proteasome is a large protein complex in the cell that is responsible for the degradation of unnecessary or damaged proteins and for post-translational regulation of the levels of many proteins via the ubiquitinylation pathway. 18 of the 60 proteins in cluster #8 are members of the proteasome (*p*-value 2.8×10^−44^) as are 22 of the 37 proteins in cluster #9 (*p*-value 2.3×10^−33^). 20 BKZ HDFs belong to cluster #8 and 9 to cluster #9. In the case of HIV infection, an active proteasome has been shown to be involved in HIV replication [45] and is necessary for the release and maturation of infectious HIV particles [46]. For example, the HIV VIF protein binds to the host APOBEC3G protein and targets it for degradation through an interaction with the proteasome [47]. This process inhibits the APOBEC3G-mediated restriction of HIV replication.

#### MHC protein complex

Of the 56 proteins in cluster #10, 10 are annotated with “MHC protein complex” (*p*-value 9.2×10^−17^). 11 predicted HDFs in the cluster are known to interact with HIV. Many of these proteins are members of the class II major histocompatibility complex; HIV protein Tat down-regulates the expression of MHC class II genes in antigen-presenting cells [48], [49].

#### Anaphase promoting complex

“Cell cycle process” is enriched in cluster #10 with a *p*-value of 5.2×10^−7^. Of the 13 proteins annotated with this process that are members of cluster #10, six proteins (ANAPC1, ANAPC4, ANAPC5, ANAPC7, ANAPC10, and ANAPC11) are subunits of the anaphase promoting complex (APC). HIV protein VPR induces G2/M arrest in order to facilitate the entry of the viral pre-integration complex into the nucleus. Studies with adenovirus and chicken anemia virus have suggested that proteins in these viruses target the APC in order to induce G2/M arrest [50]. Thus, although none of the APC proteins in this cluster are known to interact with HIV, it is possible that VPR-induced G2/M arrest may result from inhibition of the APC.

#### Nuclear pore complex

The “nuclear pore complex” is the GO term most enriched in cluster #12 (not displayed in Table 1 and in Table 2); 14 of the 18 proteins are members of this complex (*p*-value 4.7×10^−12^). Seven predicted HDFs in cluster #12, BANF1, HMGA1, NUPL2, NUP54, PSIP1, RAN, and RANBP1, interact with HIV proteins. Bushman *et al.* [10] also identified the nuclear pore, although proteins annotated to this term did not appear in a dense cluster in their analysis. The nuclear envelope is a lipid bilayer that serves as a physical barrier between the contents of the nucleus and cytoplasm. This barrier contains pores through which materials can be exchanged between the two cellular compartments. Large macromolecules require the assistance of karyopherins to pass through nuclear pores. Karyopherins bind to their cargo; after they cross the nuclear envelope, an interaction with the human RAN protein releases the bound partner. HIV has evolved to manipulate this cellular process. NUPL1 interacts with HIV VPR to mediate the docking of VPR at the nuclear envelope, a step that contributes to the nuclear import of viral DNA [51], [52]. RAN bound with GTP is known to bind to a complex of HIV protein REV and exportin 1 (CRM1) to mediate nuclear export of HIV mRNA [53], [54]. The Barrier-to-autointegration factor BANF1 is localized both to the nucleus and to the cytoplasm. It is known to be exploited by retroviruses for promoting integration of viral DNA into the host chromosome [55].

### BKZ and predicted HDF genes are differentially expressed during AIDS development in non-human primates

Since HDFs play a critical role in HIV replication [1], [2], [3], we hypothesized that some of them may have value as prognostic markers of HIV pathogenesis and of AIDS development and progression. We anticipated that both experimentally-detected (BKZ) and predicted HDFs would satisfy this hypothesis. To explore this question, we combined BKZ HDFs and predicted HDFs with DNA microarray data from a study detailing the host response to simian immunodeficiency virus (SIV) infection in African green monkeys (AGMs) and pigtailed macaques (PTMs). AGMs are natural reservoirs of SIV that do not develop AIDS, while PTMs are non-natural hosts that develop AIDS when infected with SIV. The virus replicates to the same viral load in both of these hosts. Lederer *et al.* [56] performed a longitudinal transcriptomic analysis comparing AGMs to PTMs. They analyzed the host response in the setting of acute SIV infection with the same primary isolate (SIVagm.sab92018). They studied three different tissues: blood, colon, and lymph nodes. They collected samples at 10 days and 45 days post-viral inoculation and compared each sample to a sample from the same animal pre-inoculation. For each day-tissue combination, they performed an analysis of three AGMs and three PTMs using rhesus macaque (*Macaca mulatta*) oligonucleotide microarrays. The probes in this microarray were based on the human Reference Sequence (RefSeq) collection. Thus, there is a direct mapping from these probes to human gene identifiers.

For each tissue (blood, colon, lymph node) and day (10 and 45 post infection) combination, we performed a separate ANOVA analysis, using the host system as factor, to identify genes that are differentially expressed between AGMs and PTMs. Such differentially expressed genes could potentially serve as diagnostic markers of AIDS development and progression. We constructed six lists (three tissues×two time points) of genes that were differentially expressed between AGMs and PTMs to a statistically-significant extent (*p*≤0.05). We used the one-sided version of Fisher's exact test to determine if BKZ HDFs had a significant intersection with each of these six lists. We repeated this test with the top *k* predicted HDFs, for values of *k* starting at 100 and in increments of 100. We used the method of Benjamini and Hochberg [26] to correct for testing multiple hypotheses.

**Figure 2:**
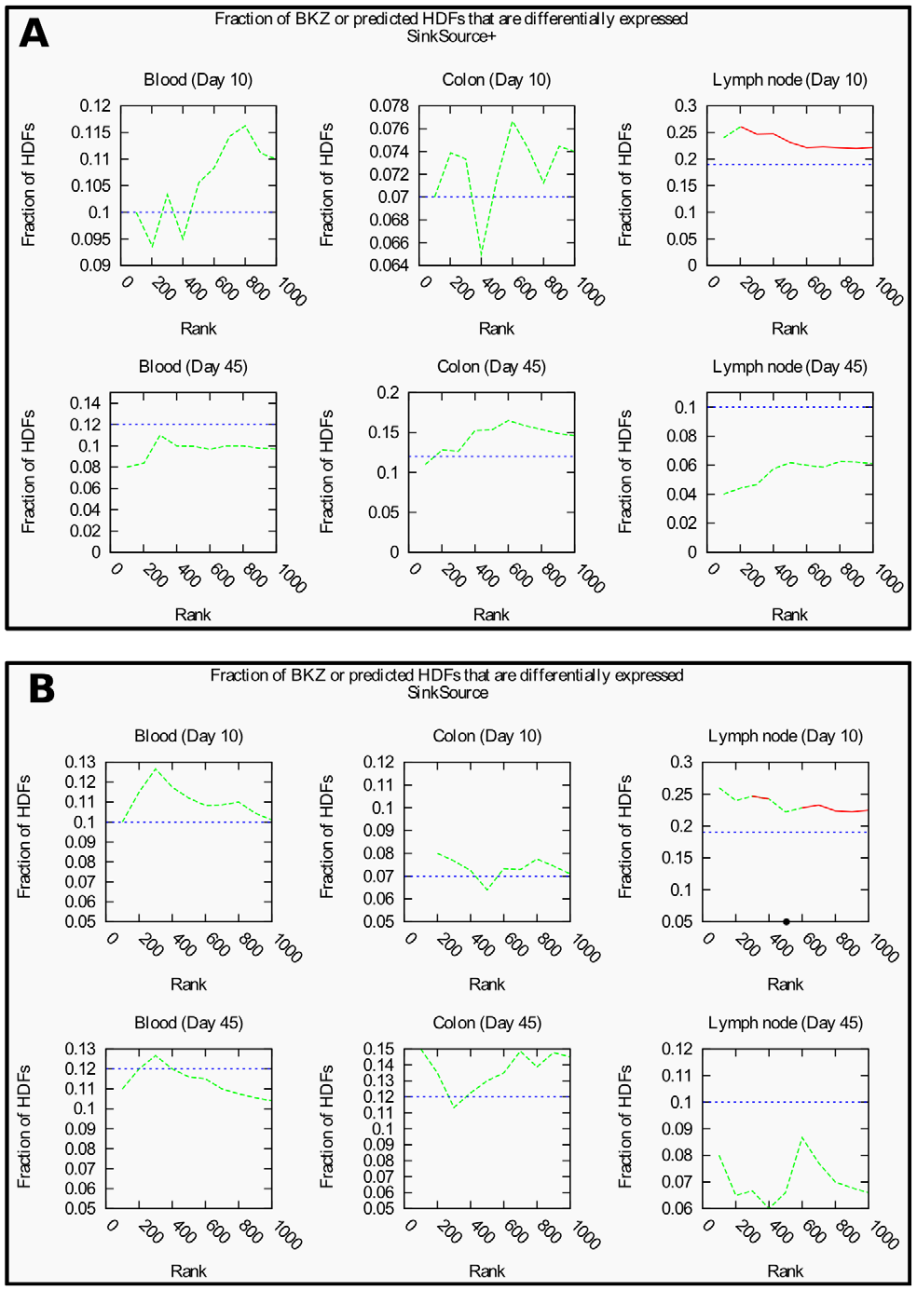
Plots of the fraction of BKZ or of predicted HDFs that are also differentially expressed in the AGM-PTM comparison: (a) SinkSource+ and (b) SinkSource. There are six plots for each algorithm, with one plot for each tissue-day combination. In each plot, the *x*-axis corresponds to the rank of a predicted HDF. At each rank *k* on the *x*-axis, the *y*-axis plots the fraction of HDFs with the top *k* ranks that are also differentially expressed. *Note that the scale of the y-axis changes from plot to plot*. The red and green curves display the results for predicted HDFs, at different prediction ranks. Red values indicate statistically significant overlaps, at the 0.05 level, between predicted HDFs and differentially-expressed genes. Green values indicate overlaps that are not statistically significant. Figures S9 and S10 plot the corresponding *p*-values. The horizontal dotted blue line in each plot denotes the overlap of BKZ HDFs with the corresponding set of differentially-expressed genes.

Figure 2 displays plots of the fraction of BKZ HDFs or of predicted HDFs that are also differentially-expressed to a significant extent in the AGM-PTM comparison; Figures S9 and S10 plot the corresponding *p*-values. Note that the plot for BKZ HDFs is a horizontal line since changing the score cutoff for predictions has no effect on BKZ HDFs. Three notable trends emerged from this analysis. First, for many tissue-day combinations, the overlap fraction for predicted HDFs was larger than the overlap fraction for BKZ HDFs. These trends were most noteworthy in day 10 lymph nodes, where the overlap ratio for predicted HDFs was larger than that for BKZ HDFs over the entire range of prediction confidence values. In particular, in day 10 lymph nodes, the overlap fraction of SS+ predicted HDFs peaked at 0.26 (53 of the top 203 predicted HDFs were also differentially-expressed in day 10 lymph nodes, *p*-value 0.01). The largest overlap for SS predicted HDFs was also 0.26 (26 of the top 100 predicted HDFs, an insignificant *p*-value of 0.07). In contrast, the overlap ratio for BKZ HDFs with genes differentially expressed in day 10 lymph nodes was 0.19 (*p*-value, 0.59). Second, none of the overlaps of BKZ HDFs with differentially-expressed genes were statistically significant, for any tissue-day combination. In contrast, *p*-values for HDFs predicted by each algorithm were statistically significant (red points in Figure 2 and Figures S9 and S10) in day 10 lymph nodes, across a wide range of prediction confidences. Third, no statistically significant overlaps appeared for predicted HDFs in blood or colon samples at any time point or in day 45 samples from lymph nodes.

We re-estimated the significance of these results after randomizing the gene expression data, by permuting each gene's *p*-values independently. This process retained the distribution of *p*-values for each gene, but randomized the associations between *p*-values and tissue-day combinations. We repeated the overlap analysis for predicted HDFs with each of 10,000 randomized gene expression data sets, for a total of 60,000 randomized tissue-day combinations. We observed only one randomized dataset for which any overlap ratio was at least as large as 0.26, the largest overlap ratio between HDFs predicted by SinkSource+ and genes differentially expressed in day 10 lymph nodes. Thus, the *p*-value of the observed overlap ratio was 1.7×10^−5^. For predictions made by SinkSource, we obtained a *p*-value of 8.3×10^−5^, for the largest observed overlap of 0.26.

Thus, we concluded that the predicted HDFs have a significant overlap with genes that are differentially expressed between AGMs and PTMs in day 10 lymph nodes, indicating that many predicted HDFs show considerably different programs of expression in the two species in response to SIV infection, especially in early time points. These data suggest that the algorithms have identified a highly responsive subset of potential HDFs, and provide strong experimental support for the prediction that these proteins are in fact HDFs. This result further suggests that viral manipulation of these host factors in lymph nodes soon after infection may have an effect on long-term pathological outcome. We used FuncAssociate to perform GO enrichment analysis on predicted HDFs that were also differentially expressed between AGMs and PTMs in day 10 lymph nodes. The terms we found were almost identical to those reported in the PPI clusters (data not shown). In summary, these results suggest that not only are HDFs critical for viral replication and infection, they may have potential value as prognostic markers to determine pathological outcome and the likelihood of AIDS development.

### Conclusions

We have used network-based approaches to predict HIV dependency factors (HDFs). Upon two-fold cross-validation, we found that combining the three experimental data sets yielded much higher precision and recall than using each data set on its own. A number of the algorithms we compared achieved both high precision and recall on cross validation. Our results suggest that global optimization techniques such as SinkSource and SinkSource+ perform slightly better than the simple guilt-by-association rule [57]. Furthermore, SinkSource+ and SinkSource had the most consistent and reliable performance. Software implementing the function prediction algorithms is available at http://bioinformatics.cs.vt.edu/~murali/software/gain. We also observed that estimating the reliability of PPIs did not confer an advantage; in fact, the cross validation results worsened slightly with edge weights (Table S2). The decrease in performance is likely to be a combination of the close proximity of HDFs within the PPI network and the high reliability of PPIs that HDFs are involved in, since the corresponding biological processes are well studied.

We found that the HDFs predicted by SinkSource+ were significantly enriched in proteins that interact with HIV proteins. On the other hand, SinkSource predicted a set of HDFs that were not significantly enriched in HIV-interacting proteins. We computed clusters within the subgraph of the PPI network that encompassed the BKZ HDFs and HDFs predicted by SinkSource+. These clusters were enriched in host cellular complexes and pathways known to be that are known to be manipulated by HIV and perturbed during HIV infection such as the spliceosome, the microtubule network, the proteasome, the mitochondrion, and nuclear import and export.

Finally, we integrated BKZ HDFs and predicted HDFs with gene expression data from a non-human primate study detailing the host response to SIV infection in non-human primates that do not develop AIDS (African green monkeys) and those that do (pigtailed macaques) [56]. We found that up to 26% of predicted HDFs are differentially expressed, when we compared their gene expression profiles in macaques to their profiles in African green monkeys. This differential expression of HDFs was time- and tissue-specific, being strongest in lymph nodes 10 days post-inoculation. These HDFs are excellent candidates for studying transcriptional programs relevant to AIDS progression in humans.

Our results support three conclusions. First, existing genomic screens are incomplete and many HDFs are yet to be discovered. The HDFs predicted by SinkSource+ may include many proteins required for HIV replication that could not have been uncovered experimentally because the predictions were not constrained to non-essential human proteins. Second, HDFs are clustered in the human PPI network and belong to cellular pathways or protein complexes that play a critical role in HIV pathogenesis and AIDS progression. Third, many HDF genes show differential expression during AIDS development in non-human primates. Thus, HDFs may play an important role in the control of initial infection and eventual pathological outcome.

It will be valuable to integrate other HIV-relevant functional genomic data with PPI networks to improve the quality and robustness of HDF prediction. Modeling the impact on off-target effects of siRNAs on false positive HDFs is also important. To date, experiments that have detected HDFs have been performed in cell lines. Approaches such as ours may help to prioritize HDFs for further experimental study in more disease-relevant models such as non-human primates. Ultimately, we anticipate that future extensions of our work may provide multiple new targets and strategies for combating HIV in humans.

Our approach is general purpose and can be applied to interpret other genome wide gene-level studies. In particular, if independent labs have conducted multiple studies to study the same biological system or phenomenon, we provide a methodology to interpret them simultaneously within the context of molecular interaction networks. Our approach can be used to ask if the studies reinforce or contradict each other and to prioritize new genes for further experimental analysis.

## Methods

### Datasets used

We downloaded all the HDF and PPI data used in this study between August and December 2008. We downloaded functional annotation data in December 2010. We used Entrez Gene IDs in all analyses.

#### HDFs (positive examples)

We gathered 275 HDFs from the study done by Brass *et al.* [1], 296 HDFs from the study done by Konig *et al.* [2] and 375 from the study done by Zhou *et al.* [3]. There were 908 unique HDFs in the union of these sets. These genes served as positive examples for our algorithm.

#### Essential genes (negative examples)

Some of our algorithms also require negative examples as input, i.e., human proteins that are not HDFs. In general, since biological datasets rarely include negative results, selection of negative examples is a challenge for many problems in computational biology that are addressed using a machine learning framework [58]. We describe one method that has proven successful in our analysis, noting that the problem of selecting appropriate negative examples is one that merits further study. By definition, HDFs are non-essential to human cells when silenced. Therefore, we used proteins that are lethal to human cells when silenced as negative examples. Since comprehensive lists of essential human genes are not available, we used human orthologs of essential mouse proteins as negative examples. Accordingly, we obtained lists of mouse proteins that are essential during prenatal, perinatal, and postnatal development from the Mouse Genome Informatics [59] database. Next, we used the InParanoid [60] database to identify human proteins orthologous to these mouse proteins. We considered a pair of proteins (one mouse, one human) to be orthologs if they were found in the same ortholog set in the InParanoid database. We identified 483 such proteins. We removed any HDF from the set of positive examples if the HDF was orthologous to an essential mouse protein. We used this approach because a gene that is essential to the organism as a whole may not be essential to a single cell. For example, En1 encodes a transcription factor essential for proper patterning of the embryo. Mice homozygous for a knockout allele die within 24 hours of birth with defects of the skeleton and nervous system. However, embryonic cells lacking this gene grow and divide and exhibit normal metabolism with only embryonic patterning being affected [61]. If this gene were silenced in a cultured cell, one would incorrectly conclude that it is not essential to the organism.

Table 3 summarizes statistics on the overlaps between HDFs and human orthologs of essential genes in mouse. The last column of the table displays the statistical significance of each overlap based on the one-sided version of Fisher's exact test, assuming that the size of the universe from which genes are selected is 20,000 (the approximate size of the siRNA libraries used in the Brass, Konig, and Zhou studies). These *p*-values are not corrected for testing multiple hypotheses. Since the smallest *p*-value is 0.013, the overlaps are statistically insignificant, at the 0.01 level.

**Table 3:** The overlap of the genes reported by each siRNA study with the set of human orthologs of essential mouse genes.

Study name	#genes	#genes that are also essential	*p*-value of overlap
Brass	275	5	0.807
Konig	296	14	0.013
Zhou	375	12	0.2
Brass, Konig, or Zhou	908	28	0.112

We acknowledge that some essential human proteins may be manipulated by HIV. As a result of this choice, some potential HDFs that interact with essential proteins may be missed by our algorithm. However, we note that the SinkSource+ algorithm, which requires no negative examples, provided predictions that overlapped substantially with the SinkSource algorithm. This result suggests that human orthologs of essential mouse genes are a suitable choice for negative examples.

#### Protein-protein interactions

We gathered human protein-protein interaction data from seven public databases, BIND, DIP, HPRD, IntAct, MINT, MIPS, and Reactome [16], [17], [18], [19], [20], [21], [22]. After removing duplicate interactions and self-interactions, we obtained a total of 71,461 interactions involving 9,595 proteins. Since many of the interactions come from high-throughput studies and since such studies are known to have numerous false positives, we applied the method of Goldberg and Roth [62] to estimate the reliability of each PPI. Under the assumption that PPI networks have the small world property, the authors argued that two interacting proteins should share many common interactors. For each PPI *(a, b)*, they counted the number of proteins that interact both with protein *a* and with protein *b*. They estimated the reliability of each interaction as the probability that *a* and *b* would have this many common interactors or more had the interactors been chosen randomly. We used the absolute value of the logarithm of this probability as a measure of the reliability of the interaction. The larger this value, the more reliable we believe the interaction is. We considered many other methods that have been used for computing the reliability of PPIs [63]. However, we decided not to use these methods since they used additional types of information, e.g., functional annotations or gene expression data, that we have used in this work to perform computational validations of our predictions.

As noted earlier, we restricted the four sets of positive examples to those proteins that participated in at least one interaction in the PPI network. Table 4 lists the number of genes in each study and the size of the overlap with the PPI network.

**Table 4:** The number of genes in each set and the number in each set that are also in the PPI network.

Study name	#genes	#genes that are also in the PPI network
Brass (B)	275	157
Konig (K)	296	199
Zhou (Z)	375	215
Brass, Konig, or Zhou (BKZ)	908	545
Essential genes	483	373

### The SinkSource algorithm

We modeled the human protein interaction network as an undirected graph *G = (V, E)*, consisting of a set *V* of nodes (i.e., proteins) and a set *E* of edges (i.e., interactions). We used *w_uv_* to denote the weight of the edge 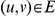, computed as described earlier. We partitioned *V* into three subsets 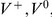 and *V^−^* as follows: *V^+^* was the set of HDFs (positive examples), *V^−^* was the set of human proteins orthologous to essential mouse proteins (negative examples), and *V^0^* was the remaining set of nodes (unknown examples). For each node *v*∈*V^0^*, our goal was to assess whether *v* should be a member of *V^+^* or *V^−^*. We did so by computing a function 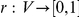 that is “smooth” over *G*. Specifically, we set *r(v) = 1* for every node *v*∈*V^+^*, *r(v) = 0* for every node *v*∈*V^−^*, and required that *r* minimize the function
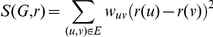
Minimizing *S(G, r)* enforces the smoothness of *r* in the sense that the larger the weight of an edge *(u, v)*, the closer in value *r(u)* and *r(v)* must be. The function *S(G, r)* is minimized when, for each node *v*∈*V^0^*,
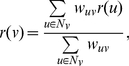
(1)where *N_v_* is the set of neighbors of node *v* [64]. The right-hand side of this equation can be split into two parts: one corresponding to contributions to *r(v)* from neighbors in *V^0^* and the second to a constant contribution from neighbors in *V^+^* and *V^−^*. Let *r^0^* denote the vector of values taken by the function *r* at the nodes in *V^0^*. Let *M* denote the square matrix, where 
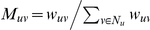
, for every 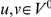. We see that *r^0^* satisfies the equations *r^0^ = Mr^0^+c*, where *c* is a vector denoting contributions from *V^+^* and *V^−^*. We computed *r^0^* by initializing it to 0 for each node 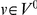 and repeatedly applying the operation *r^0^ = M r^0^+c*. This process is known to converge [64], yielding a value of *r^0^ = (I−M)^−1^c*, where *I* is the identity matrix. The matrix *M* is sparse, being the adjacency matrix of a PPI network. Therefore, this iterative approach is efficient in practice.

### Other algorithms

We implemented six other algorithms for the purpose of comparison. The first two algorithms use both positive and negative examples. The other four algorithms do not use negative examples for making predictions, avoiding the uncertainties associated with choosing an accurate set of negative examples. We used both types of algorithms in order to assess the impact of our choice of negative examples on the cross validation results. Table 5 summarizes these algorithms.

- The Local algorithm (also called “Guilt-by-association” in the literature) initializes *r(v)* = 0 for each node 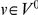 and applies equation (1) exactly once to each node 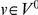.
- The Hopfield network algorithm [13] sets *r(v) = 1* for every node *v*∈*V^+^*, *r(v) = −1* for every node *v*∈*V^−^*, and initializes *r(v) = 0* for every node in *v*∈*V^0^*. The algorithm repeatedly applies a modified form of equation (1), by setting *r(v)* to be the sign of the right hand side of equation (1). Thus, it restricts *r(v)* to take the value 1 or −1. This process is also known to converge [13].
- We used a modified version of the SinkSource algorithm that does not need negative examples. Specifically, we set *r(v) = 1* for every node *v*∈*V^+^* as before. We added an artificial node *t* to *G*, fixed *r(t) = 0*, and connected each node in *V^0^* or *V^−^* to *t* using an edge of weight λ. The node *t* serves as an artificial negative example. The value computed at every node *v* in *V^0^* or *V^−^* satisfies the equation
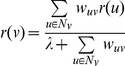
Note that the parameter λ appears in the denominator. We called the modified algorithm SinkSource+. We ran this algorithm for seven different values of λ ranging over four orders of magnitude: 0.01, 0.1, 0.5, 1, 2, 10, and 100.
- The Local+ algorithm is identical to Local, except that Local+ does not use negative examples.
- The FunctionalFlow [14] algorithm does not use negative examples. The algorithm runs in phases. Each positive example has an infinite reservoir of fluid in all phases. Each unknown example has an empty reservoir at phase 0. In each phase, fluid flows along each edge from the node with a larger reservoir to the node with a smaller reservoir. The flow equations are formulated as follows:
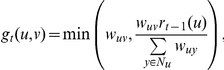
where 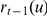 is the reservoir level at node *u* after phase *t−1* and 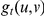 is the flow from node *u* to its neighbor *v* in phase *t*. This flow is defined only when 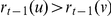; otherwise, it is 0. The algorithm updates each node's reservoir level in phase *t* based on the flow equations. The total inflow into a node over all phases represents the confidence with which the node is predicted to be an HDF. This algorithm needs the number of phases as input. As suggested by the authors, we used half the diameter of the human PPI network; the diameter was 14, so we used 7 phases. We also ran the algorithm for one, three and five phases, to assess the effect of the number of phases on the results.
- The PRINCE algorithm [15] is another flow based algorithm, developed for the task of prioritizing disease-related genes in the context of a protein interaction network. It is naturally applicable for the task of predicting HDFs. This algorithm uses *s(v)* to represent the prior information for each node *v*. Specifically, *s(v) = 1* for every node *v∈V^+^* and *s(v) = 0*, otherwise. This algorithm computes a value *r(v)* for every node *v* in *V* such that *r(v)* is close to the value at the neighbors of *v* and, for the nodes in *V^+^*, close to the initial value *s(v)*. PRINCE uses the update equation
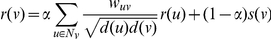
where *d(u)* is the total weight of the edges incident on node *u* and α is a parameter between 0 and 1 that trades off the relative contribution of the neighbors against prior information. We ran PRINCE for nine distinct values of α between 0.1 and 0.9 in steps of 0.1.

**Table 5:** The seven algorithms tested, whether they use negative examples, the parameters they use, and the values of the parameters tested.

Algorithm	Uses negative examples	Parameters	Values tested
SinkSource	Yes	None	
Local	Yes	None	
Hopfield	Yes	None	
Local+	No	None	
SinkSource+	No	λ = weight of edges incident on artificial negative example	0.01. 0.1, 0.5, 1, 2, 10, and 100
FunctionalFlow	No	Number of phases	1, 3, 5, 7
PRINCE	No	α = trade-off between contributions from neighbors and prior information	0.1 to 0.9 in steps of 0.1

Although SinkSource+, Local+, FunctionalFlow, and PRINCE do not use negative examples when making predictions, we used negative examples when computing the performance of these algorithms on cross validation in order to count the number of true negatives and false positives.

#### Qualitative comparison of SinkSource+, PRINCE, and FunctionalFlow

All three algorithms do not use negative examples. However, they have important differences. SinkSource+ and PRINCE are more akin to each other than to FunctionalFlow: they compute *r(v)* as the sum of contributions from the neighbors of *v* in such a way that *r(v)* is smooth over G. They differ from each other in the way they handle edge weights and information from positive examples. The most important difference between PRINCE and SinkSource+ is that PRINCE allows the value *r(v)* to change even for nodes in *V^+^*, whereas SinkSource+ fixes these values at 1. In contrast, FunctionalFlow does not explicitly set out to compute a smooth value of *r(v)*. Moreover, both PRINCE and SinkSource+ are guaranteed to converge, but FunctionalFlow must be stopped after a user-specified number of rounds.

### Computing enriched functions

A number of approaches are available for computing GO terms enriched in lists of genes [23], [65], [66], [67]. Since BKZ HDFs are unordered while predicted HDFs can be ranked by confidence, we used the FuncAssociate software [23], which can take both unordered and ordered lists of genes as input. For an ordered list of genes, FuncAssociate analyses each one of the list's prefixes, and reports results for the prefix with the smallest *p*-value. It asks if the genes annotated by each GO term have surprisingly low ranks in the ranked list. The final p-value computed by FuncAssociate can be informally interpreted as the probability that a given overlap between a GO term and a ranked list of genes could be observed if the genes were ranked randomly. Note that FuncAssociate operates solely on the ranked list of genes and the GO annotations. It does not utilize a network. Details on how FuncAssociate operates are provided at http://llama.mshri.on.ca/FuncAssociate_Methods.html.

To determine enriched GO functions in each cluster computed by MCODE, we did not associate any weights with the proteins, since MCODE had already incorporated protein weights. We used an in-house implementation of the Ontologizer [68] to compute enriched GO terms. We chose the Ontologizer because it accounts for annotation dependencies that arise from GO's true path rule. We retained only those functions for which the *p*-value is at most 0.05, after accounting for multiple hypothesis testing using the method of Benjamini and Hochberg [26].

### Modifying MCODE to compute PPI clusters

We modified MCODE to multiply internally-computed node weights with externally-defined node weights. For our application, we supplied the SinkSource+-derived confidence as the weight of a predicted HDF. For every BKZ HDF, we defined its weight as 1. By imposing these externally-defined weights, we aimed to bias MCODE towards finding dense subgraphs in the vicinity of BKZ and SS+ predicted HDFs. Therefore, we included all SS+ predictions together with their confidence levels in the network and used the ability of MCODE to utilize the confidence levels to identify high confidence clusters.

## Supporting Information

Figure S1 Precision-recall curves for Functional Flow with 1, 3, 5, and 7 phases on the BKZ dataset with the unweighted PPI network. As shown in the figure, the cross validation performance of Functional Flow on the BKZ dataset with the unweighted network does not vary much as the number of phases increases.
(EPS)

Figure S2 Precision-recall curves for SinkSource+ with different values of λ on the BKZ dataset with the unweighted PPI network. The cross validation performance does not change substantially when we change λ over four orders of magnitude.
(EPS)

Figure S3 Precision-recall curves for PRINCE with different values of α on the BKZ dataset with the unweighted PPI network. The cross validation performance does not change substantially when we change α from 0.1 to 0.9 in steps of 0.1.
(EPS)

Figure S4 Performance of the algorithms on the weighted PPI network. (a) Histograms of area under precision-recall curve for all algorithm-dataset combinations for the weighted PPI network. Each group of vertical bars corresponds to one algorithm. Error bars indicate one standard deviation from the mean, computed over 10 independent runs of 2 fold cross validation. Algorithm abbreviations: Hopfield (H), Local (L), SinkSource (SS), FunctionalFlow with 1 phase (FF 1), FunctionalFlow with 7 phases (FF 7), Local without negative examples (L+), SinkSource without negative examples (SS+), and PRINCE (P). Dataset abbreviations: Brass (B), Konig (K), Zhou (Z), Brass or Konig or Zhou (BKZ). (b) Precision-recall curves for the SinkSource algorithm on the four datasets with the weighted PPI network. At each value of recall, error bars indicate one standard deviation in the value of precision. (c) Precision-recall curves for the SinkSource+ algorithm on the four datasets with the weighted PPI network. (d) Precision-recall curves for all algorithms on the BKZ dataset with the weighted PPI network.
(EPS)

Figure S5 Precision-recall curves for Local+ and FunctionalFlow with 1 phase on the BKZ dataset with the unweighted PPI network. These results show that there is high variation in the performance of Local+ and FunctionalFlow with 1 phase.
(EPS)

Figure S6 Overlap between HDFs predicted by SinkSource+ and by SinkSource. The *x*-axis represents the *k* highest confidence HDFs predicted by SinkSource+ and by SinkSource. At each value of *k* on the *x*-axis, the *y*-axis represents the Jaccard coefficient between the *k* highest confidence HDFs predicted by SinkSource+ and the *k* highest confidence HDFs predicted by SinkSource.
(EPS)

Figure S7 Comparison of prediction ranks for SinkSource+ with different values of λ. Each point on each plot represents one gene. Each plot compares the prediction confidence with λ = 1 for a gene (*x*-axis) to the confidence for that gene with another value of λ (*y*-axis).
(TIFF)

Figure S8 Overlap between HDFs predicted by SinkSource+ for different values of λ. On each plot, the *x*-axis represents the *k* highest confidence HDFs predicted by SinkSource+ with λ = 1, for different values of *k*. At each value of *k* on the *x*-axis, the *y*-axis represents the Jaccard coefficient between the *k* highest confidence HDFs predicted by SinkSource+ (λ = 1) and the *k* highest confidence HDFs predicted by SinkSource+ for another value of λ.
(EPS)

Figure S9 Plots of *p*-values for overlap of BKZ or of SinkSource+ predicted HDFs with genes that are differentially expressed in the AGM-PM comparison. Each plot corresponds to a tissue-day combination. In each plot, the *x*-axis corresponds to the rank of a predicted HDF and the *y*-axis to the absolute value of the base-10 logarithm of the *p*-value corresponding to the fraction of HDFs that are also differentially expressed. Note that the scale of the *y*-axis changes from plot to plot. The red and green curves display the results for predicted HDFs, at different prediction ranks. The red curve corresponds to those rank cutoffs for which the *p*-value of Fisher's exact test is at most 0.05, whereas the green curve corresponds to *p*-values>0.05. The horizontal blue line in each plot denotes the overlap of BKZ HDFs with the corresponding set of differentially-expressed genes. Note that some plots are empty because all the *p*-values evaluate to 1, after correction for multiple hypothesis testing.
(EPS)

Figure S10 Plots of *p*-values of overlaps of BKZ or of SinkSource predicted HDFs with genes that are differentially expressed in the AGM-PT comparison. See the caption for Figure S8 for details.
(EPS)

Table S1 AUPRC values for all algorithms and all datasets for the unweighted protein interaction network. The columns in the table are (a) Experiment: a mnemonic string describing the dataset, algorithm, parameters, and PPI network, (b) Algorithm: an abbreviation for the algorithm, (c) Mean AUPRC, (d) Std dev AUPRC, (e) Mean AUC, and (f) Std dev AUC.
(XLS)

Table S2 AUPRC values for the weighted PPI network. The columns are the same as in Table S1.
(XLS)

Table S3 Comparison of FuncAssociate results between BKZ HDFs and HDFs predicted by SinkSource+. For each function in this table, the column titled “N (BKZ)” contains the number of BKZ HDFs annotated with the function, and the column titled “X (BKZ)” contains the number of genes annotated with the function. For SinkSource+, the corresponding columns and the column “M (SinkSource+)” refer to the most statistically-significant prefix of the SinkSource+ predictions ordered by rank. See the FuncAssociate documentation (http://llama.mshri.on.ca/funcassociate/documentation) for details. Note that a p-value of 0 only means that the observed statistic was never seen in the permuted data. Since we ran FuncAssociate with 1,000 permutations, a *p*-value may be taken to a value less than 0.001.
(XLS)

Table S4 Comparison of FuncAssociate results between HDFs predicted by SinkSource+ and by SinkSource. Columns are similar to those in Table S3, except that this table compares FuncAssociate results for SinkSource+ with FuncAssociate results for SinkSource.
(XLS)

Table S5 Statistically-significant overlaps of BKZ HDFs with clusters computed by MCODE. Columns are (a) the ranking of the cluster by #PPIs, (b) the *p*-value of the overlap between BKZ HDFs and proteins in the cluster, (c) the #BKZ HDFs in the cluster, (d) the #proteins in the cluster, and (e) the fraction of proteins in the cluster that are BKZ HDFs.
(XLS)

Table S6 Annotated predictions made by the SinkSource+ algorithm on the BKZ dataset with the unweighted PPI network, sorted in decreasing order of prediction confidence (column (d)). The columns in the table are (a) Entrez Gene id, (b) gene symbol, (c) whether the gene is a BKZ HDF or not, (d) prediction confidence (1 for BKZ HDFs), (e) rank of the prediction (0 for BKZ HDFs), (f) whether the gene is known to interact with HIV or not, (g) MCODE cluster the gene belongs to, (h) full name for the gene, and *p*-value of differential expression of the gene in the study by Lederer *et al.* in (i) Lymph node (Day 10), (j) Lymph node (Day 45), (k) Colon (Day 10), (l) Colon (Day 45), (m) Blood (Day 10), (n) Blood (Day 45). Note that some BKZ HDFs overlap with human orthologs of essential genes in mouse. SinkSource+ treats these genes as unknown examples. Therefore, these genes have an entry of “BKZ” in column (c) and a prediction confidence less than 1 in column (d).
(XLS)

Table S7 Human PPIs in each MCODE cluster. The columns in the table are (a) MCODE cluster id, (b) Entrez Gene id of interactor 1 (c) gene symbol of interactor 1, (d) Entrez Gene id of interactor 2, and (e) gene symbol of interactor 2.
(XLS)
