# Supplementary figures and images for: Network-Based Prediction and Analysis of HIV Dependency Factors
Source: PLoS Comput Biol. 2011 Sep 22;7(9):e1002164. doi: 10.1371/journal.pcbi.1002164 (PMC3178628; doi:10.1371/journal.pcbi.1002164)

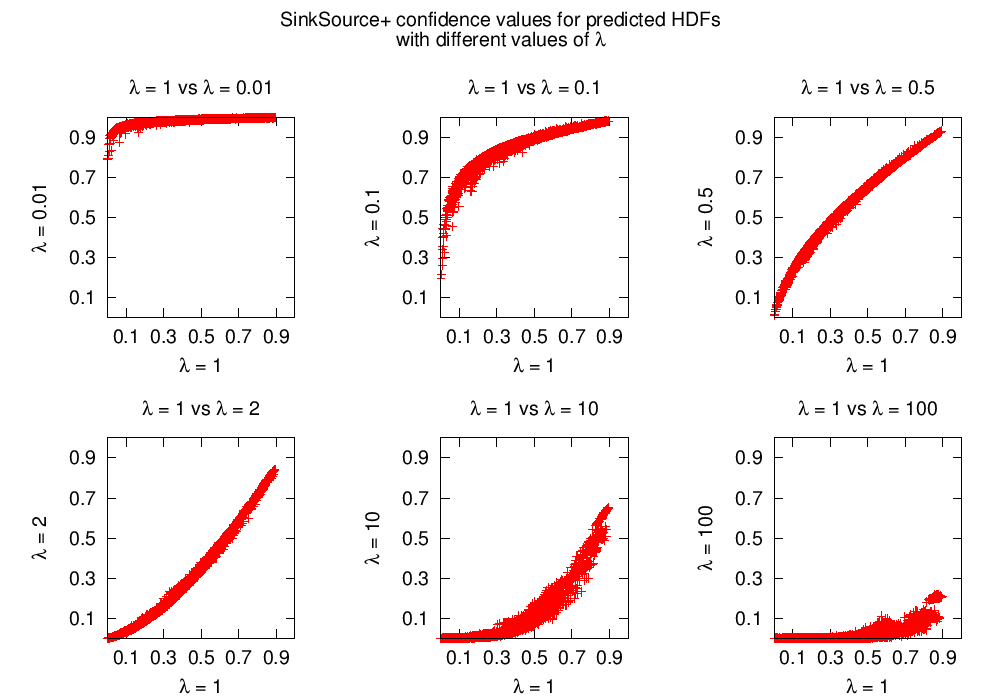

Supplement: Figure S7 — Comparison of prediction ranks for SinkSource+ with different values of λ. Each point on each plot represents one gene. Each plot compares the prediction confidence with λ = 1 for a gene (x-axis) to the confidence for that gene with another value of λ (y-axis). (TIFF) [file pcbi.1002164.s007.tiff]
